# 12 Plagues of AI in Healthcare: A Practical Guide to Current Issues With Using Machine Learning in a Medical Context

**DOI:** 10.3389/fdgth.2022.765406

**Published:** 2022-05-03

**Authors:** Stephane Doyen, Nicholas B. Dadario

**Affiliations:** ^1^Omniscient Neurotechnology, Sydney, NSW, Australia; ^2^Robert Wood Johnson Medical School, Rutgers University, New Brunswick, New Jersey, NJ, United States

**Keywords:** artificial intelligence, machine learning, deep learning, medical software, cloud computing, neural network, medicine

## Abstract

The healthcare field has long been promised a number of exciting and powerful applications of Artificial Intelligence (AI) to improve the quality and delivery of health care services. AI techniques, such as machine learning (ML), have proven the ability to model enormous amounts of complex data and biological phenomena in ways only imaginable with human abilities alone. As such, medical professionals, data scientists, and Big Tech companies alike have all invested substantial time, effort, and funding into these technologies with hopes that AI systems will provide rigorous and systematic interpretations of large amounts of data that can be leveraged to augment clinical judgments in real time. However, despite not being newly introduced, AI-based medical devices have more than often been limited in their true clinical impact that was originally promised or that which is likely capable, such as during the current COVID-19 pandemic. There are several common pitfalls for these technologies that if not prospectively managed or adjusted in real-time, will continue to hinder their performance in high stakes environments outside of the lab in which they were created. To address these concerns, we outline and discuss many of the problems that future developers will likely face that contribute to these failures. Specifically, we examine the field under four lenses: approach, data, method and operation. If we continue to prospectively address and manage these concerns with reliable solutions and appropriate system processes in place, then we as a field may further optimize the clinical applicability and adoption of medical based AI technology moving forward.

## Introduction

The powerful applications of artificial intelligence (AI) have long been promised to revolutionize the healthcare field. AI has been met with a surge of interest in the scientific and medical communities due to the increasing number of patients receiving healthcare services and the concomitant increases in complexity of data, which is now available, but often uninterpretable by humans alone. These technologies demonstrate the ability to identify malignant tumor cells on imaging during brain surgery (1), unravel novel diseases into explainable mechanisms of viral mutations for therapeutic design (2), predict the progression of neurodegenerative diseases to begin earlier treatments (3), and assist with the interpretation of vast amounts of genomic data to identify novel sequence patterns (4), among a number of many other medical applications. Ultimately, the applications of AI in medicine can generally be grouped into two bold promises for healthcare providers: (1) the ability to present larger amounts of interpretable information to augment clinical judgements while also (2) providing a more systematic view over data that decreases our biases. In fact, one could argue that improving our ability to make the correct diagnoses if given an opportunity can be seen as a key duty and moral of the medical field in principle (5). Given this enormous potential, it is unsurprising that Big Tech companies have matched the enthusiasm of scientific experts for AI-based medical devices by investing substantial efforts and funding in their product development over recent years (6). Unfortunately, despite these exhilarating observations and promises, we have yet to truly harness the full potential of AI as a tool in current practices of medicine.

**Figure 1:**
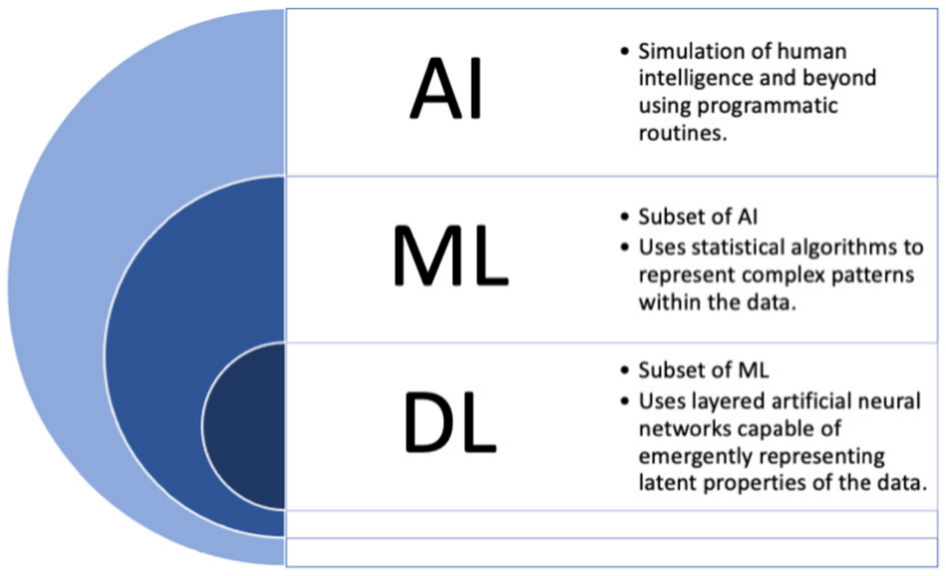
The umbrella of “AI”. AI, artificial intelligence; DL, deep learning; ML, machine learning.

Many loosely utilize the label “AI” for the medical applications described above, but providing more focused definitions for a few important terms may be beneficial moving forward in this work. The term “AI” in fact more broadly refers to the idea of machines being able to intelligently execute tasks in a manner similar to human thinking and behavior. To date, such applications are not currently found in the field. Instead, applications where machines learn from data can more accurately be labeled as machine learning (ML) based solutions. For simplicity, we will use these two terms interchangeably throughout the current manuscript, and outline commonly used terms in Figure 1 which are considered under the umbrella of “AI.”

While not newly described, ML based applications have re-surfaced interest in the medical community with the rise of the recent SARS-CoV-2 pandemic (2). International collaborative efforts from Data Scientists have attempted to take advantage of differences in disease prevalence across the world as a way of utilizing early access to data to improve the quick diagnosis and prognostication of patient outcomes in soon-to-be overwhelmed hospitals. In fact, novel approaches had immediately begun to tackle concerns on vaccine developments early in the pandemic according to possible viral mutations that allow escape from the human immune system. By framing SARS-CoV-2 protein sequence data in the context of linguistic rules used in the human natural language space, ML algorithms may be able to present to us *interpretable* mechanisms of how a virus mutates while retaining its infectivity, similar to how a word change in a human language may dramatically alter the meaning of a sentence without changing its grammar (2). Indeed, thousands of exciting models with increasing collaboration and data sharing had been immediately proposed throughout the pandemic to improve the prognostication and clinical management of COVID-19 patients (7, 8). While few clinical applications were found to demonstrate a true clinical impact on patient outcomes for the current pandemic, it is important to note that ML applications have elsewhere demonstrated important clinical applications in similar contexts (9), such as for improving the allocation of limited resources (10), understanding the probability of disease outbreak (11), and the prediction of hospital stay and in-hospital mortality (12). Therefore, while these models continue to demonstrate enormous potential to manage large scale clinical scenarios, further work is still necessary to ensure they can be quickly and effectively leveraged for clinical translation, a concern which has been encountered in the field previously.

Leading digital companies have often pioneered the implementation and excitement of AI technologies in current medical practices. IBM Watson for Oncology took years of advanced development and training with physicians from Memorial Sloan Kettering, ultimately teaming up with the MD Anderson Cancer Center in 2013, another leading cancer center in the United States, with promises of improving oncological diagnoses and therapy decision making. However, this collaboration was terminated by MD Anderson in 2017 due to failure of meeting their oncological and patient goals with this novel software (13, 14). Many believe increasing technical advancements in computational abilities will reinvigorate the potential horizons and trust of these technologies in the medical field moving forward. A new deep learning system made by Google promises to detect 26 skin conditions with accuracy comparable to US board-certified dermatologists (15), yet recent work has already come under substantial controversy due to underperformance based on underlying inter-individual differences in demographics, such as gender and skin color (16). Nonetheless, recent public data continues to demonstrate increased interest in medical AI software as seen with continued surges in AI investments in drug design and discovery, attention in large governmental plans, and focuses on AI in scientific careers for medical applications compared to each previous year (17).

The limitations mentioned above are not to say that we should abandon the field of AI-based medical technologies as a whole. The ability for human intuition alone to map the brain for instance, would take insurmountable amounts of time and effort compared to that which is now possible with AI-based technology (18–20). As original goals previously hoped that these technologies would resemble human based intelligence, it is important to remember *To Err is Human*. Instead, as with all emerging fields, further work is necessary to optimize the clinical applicability of these tools as we move forward to minimize unnecessary errors and harm. Unfortunately, there is limited information available in the literature that presents a clear guide of the common problems that will be encountered with AI-based software, and more specifically how to overcome them moving forward in medical practices. Such a gap possibly reflects the separation of expertise and focus between medical professionals and data scientists all together.

**Figure 2:**
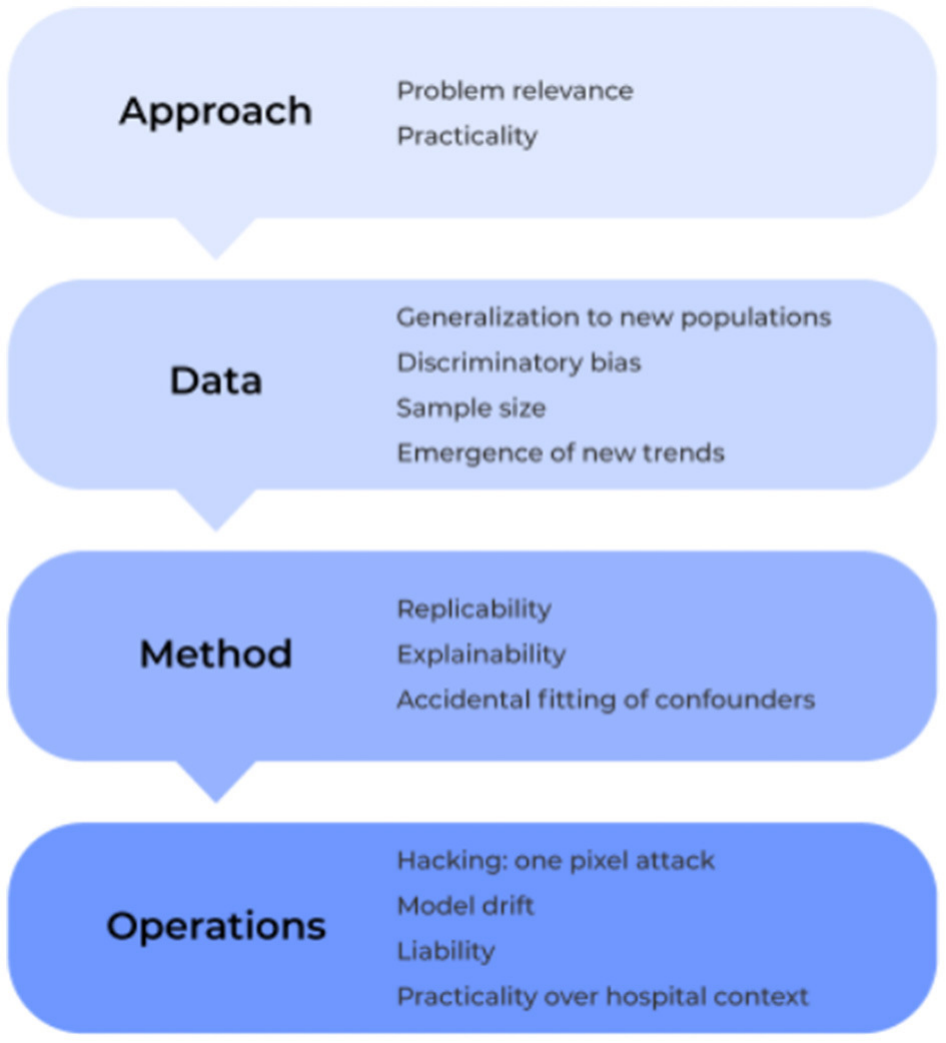
The 12 plagues of AI in healthcare. The development of AI software and deployment into the medical community can be generally grouped into four processes: approach, data, method, and operation. In each component, a number of common problems can be encountered which must be prospectively considered or managed in real-time to ensure efficient and accurate model performance in high-risk medical environments. Here, we show the most common pitfalls of AI-based medical solutions within these components and discuss them throughout the current manuscript.

To address this gap, we provide a clear guide below on the most common pitfalls to medical AI solutions based on the previous literature. Specifically, we examine the field under four lenses: approach, data, method and operation (Figure 2). Within these core components, we outline a number of different issues that must be considered in the development and application of machine learning technologies for medical based applications, and we elucidate how to identify, prevent, and/or solve them moving forward to ultimately create safer, more trustworthy, and stronger performing models.

## Approach: What Is the Plan?

### Problem 1. Relevance: Cutting Cake With a Laser

**Figure 3:**
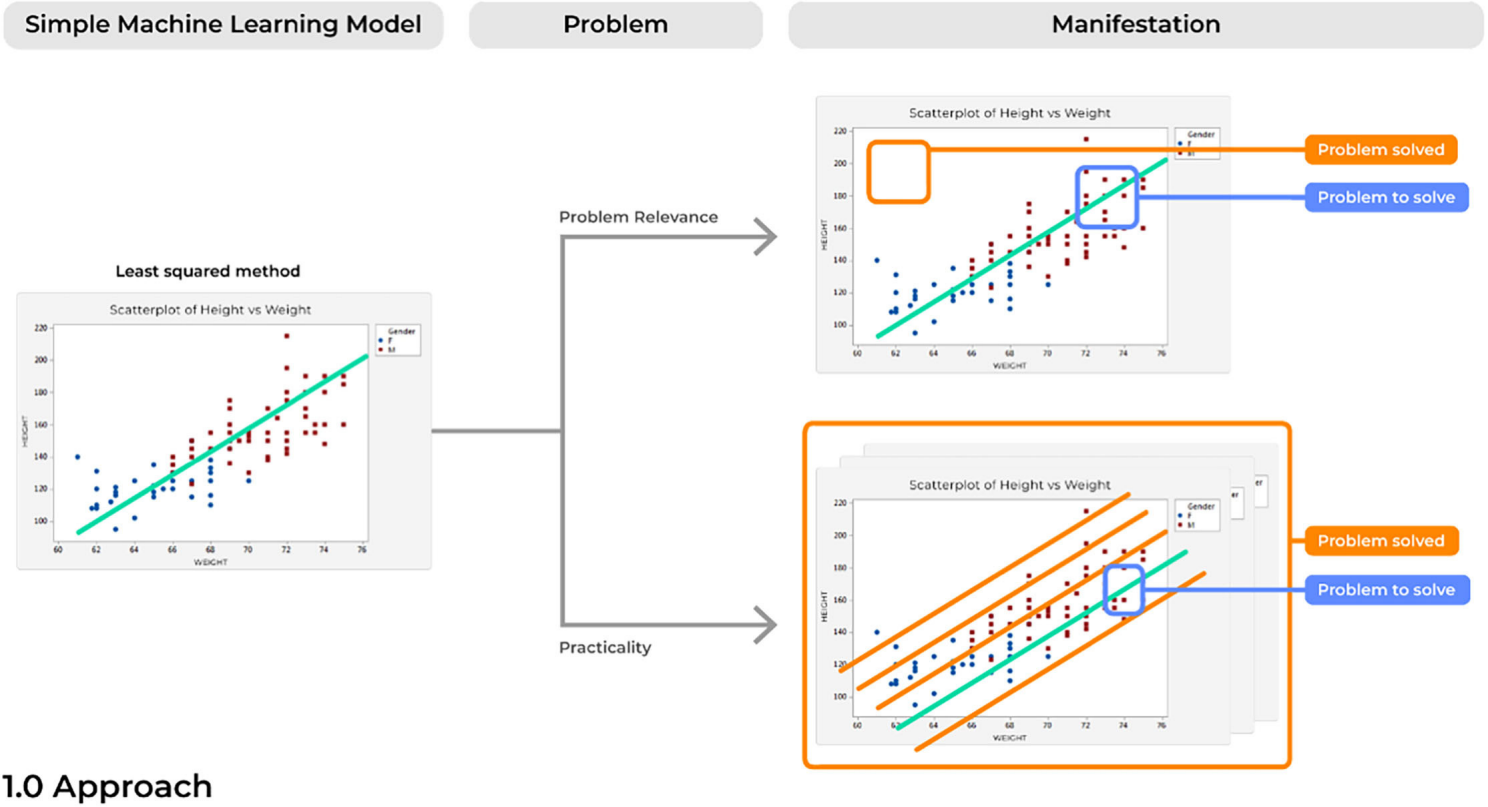
Concerns with the approach to ML product development.

Due to a rise in the amount of open-source and advanced statistical software, it has become increasingly easy to develop highly elegant and powered computational models. However, when created with only technical solutions in mind, models can easily be created to solve an non-existent or irrelevant problem. In turn, the ultimate users of the technology, such as the practicing physician in the clinic, will have no interest in the solution being answered by a specific model. Problems with a model's relevance can render even the most elegant application of data science irrelevant (Figure 3).

A common example of this can be seen with the increased ability we now have to detect mental illness based on improved and publicly available maps of the brain connectome (21, 22). If a ML specialist received a dataset including data on patients with Schizophrenia, their first thought may be to build a model to detect Schizophrenia. However, current medical practices are already highly capable of such detection and would rather see other issues provide more fruitful avenues for ML applications, such as predicting modulatory treatment responses for Schizophrenia (23). Therefore, viewing such issues from a pure computer science or statistical standpoint is inherently hindering the potential for a current project.

Instead, the ultimate users of the technology should be included at the very beginning of the development of the model. Research and Development goals should be grounded in what has already been suggested in the medical field as important avenues of future work for clinical improvements. For instance, rather than attempting to predict an illness which can already be clearly identified in clinical practice, ML tools can reduce the complexity of patient information presented in a specific pathological states (24, 25) and then present statistical irregularities that can be used by physicians to make more informed decisions for clinical treatment (26, 27). Ultimately, it must be remembered that AI is a powerful *tool* that can be leveraged to answer a difficult questions, the usefulness of the tool is a function of the appropriateness of the question being asked.

### Problem 2. Practicality: Not Everyone Has a Cyclotron

Similar to the concerns of *relevance* mentioned above, advances in ML abilities have also created concerns of *practicality*. This refers to building a model which has limited practical applications in the environment of interest due to logistical constraints that were not considered outside of the environment that the model was originally created in, such as requiring more computation than necessary or that which can be feasibly run in a clinic. Reasonable solutions must commonly consider the current state of the field and the technical constraints of the model proposed.

To create and train advanced ML models, disparate data is often harmonized from varying sources and formats to create a single large dataset of valuable information. Data harmonization is particularly important in medicine given that small amounts of data on specific topics are often managed by acquiring data from varying records and from a variety of centers, all of which also commonly utilize different electronic health record (EHR) systems (28). Therefore, to build a model that aims to compare specific patients to a template/control group, algorithms must first harmonize large amounts of a patient's data from varying datasets to perform a comprehensive comparison. However, to do this, many algorithms require datasets to be harmonized as *large cohorts*. Unfortunately, outside of the lab, these methods can become impractical when single (*N* = 1) patients present to the clinic. Therefore, these algorithms can fail unless additional practical solutions are present. Importantly, improved super computers may hold the computing power to execute a highly complex model in a lab for a single patient, but a physician may not be able to analyze a patient's data with these algorithms on a less powerful hospital-issued laptop in a time critical environment where it must perform at the highest level. On that same note, it is easy to optimistically consider the intricate problems that can be tackled with ML in the future due to the improved abilities of modern computational algorithms to digest highly complex data; however, medical data is often not available or too limited for specific topics at the current time. When just utilizing the limited available data obtained specifically from academic studies to train a model, illusory results may be obtained given these data are cleaner than that which would be obtained from the actual target field. Therefore, investing time in an approach that is too far into the future may inherently cause difficulties in model preparation due to the lack of available feedback from the clinical field of interest.

To prospectively mitigate problems of *practicality*, implementation should always be considered from the very start of any analytical solution. This will prevent any waste of Research and Development efforts given that alternative solutions were already considered. A notable increase in recent efforts has been put forth by cloud providers and hardware manufactures to provide development frameworks which bridge the gap between an approach to a problem and the hardware to support it (29). Ultimately, including the intended users in the initial development stages will also provide insight on *practicality* during model development as the goal environment will have already been considered.

## Data: What Are We Using and for What Purpose?

### Problem 3. Sample Size: Looking at the World Through a Keyhole

**Figure 4:**
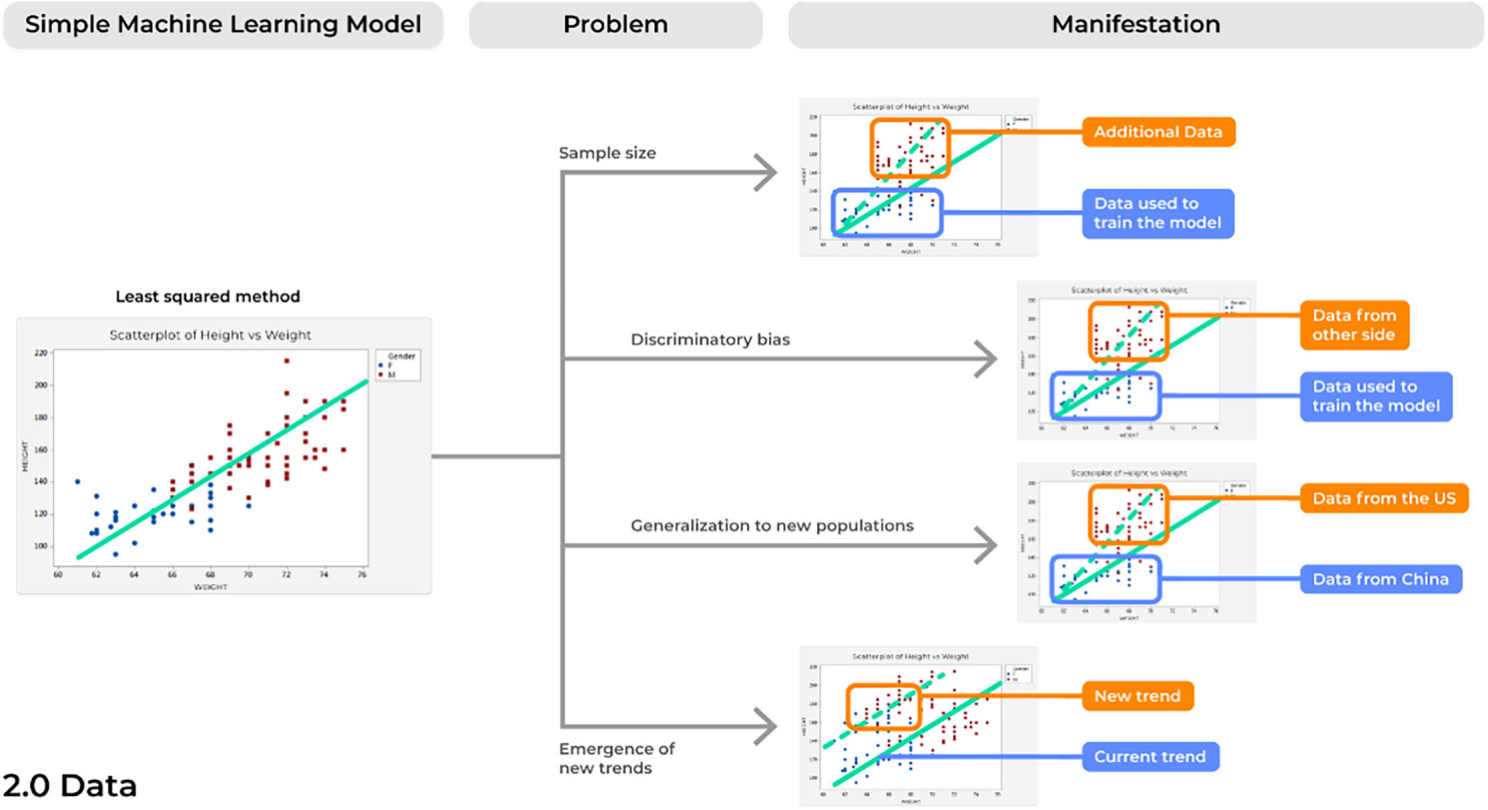
Concerns with the data a model is being trained and tested on.

A concern inherent to most analytical solutions includes issues of sample size. When creating and training a model using a limited sample size, inflated results may be demonstrated when actually testing it against a control sample. Subsequently, when introduced into a different, clinical environment, the model's accuracy can lessen, and it can fail. This is because the small sample size may cause a model to present results that are merely due to chance. Consideration of this is paramount to acquire reliable results (Figure 4).

In an age of increasingly available data, ML algorithms must be trained on adequately sized and diverse datasets. While this will likely require the harmonization of data from multiple centers, this point is imperative if we are to believe our models are robust enough to consistently provide reliable results at different time points and in a number of different environments. A recent systematic review assessed 62 comprehensive studies detailing new ML models for the diagnosis or prognosis of COVID-19 utilizing chest x-rays and/or computed tomography images and found a common problem among studies was in the limited sample size utilized to train models (8), with other reviews noting similar observations (30). Amongst the 62 models assessed, more than 50% of diagnostic focused studies (19/32) utilized <2,000 data points. Unlike rare brain tumors which may require years of small amounts of data collection, there have been more than 2 million cases of COVID-19 to date (31). Therefore, it is unsurprising that developing a model to understand a complex biological phenomenon on only 2,000 data points can lead to unreliable results in the intended environment (8).

As in all aspects of medical research, newer ML models must be trained on a large and diverse dataset to provide reliable results. A clear solution is to implement an active and ongoing effort to acquire more data from a variety of sources. ML models, unlike physical medical devices, can be continually updated and improved to provide more reliable and accurate performances. As more data becomes available, models can be retrained with larger sample sizes and then updated for the best performance in the field. Ultimately, this may require a model to be pulled from a clinical practice, retrained and tested based on more data which is now available, and then placed back into the environment of interest. However, with the increased demand for more data, one must consider the possibility that an ML modeler may acquire and include data for which they have no patient consent. Once the new data is incorporated into the model and the model is input into the field, it is very difficult to identify which data was utilized. A possible solution to these patient consent concerns with increasing data is implementing in each image a certificate stating patient consent, such as through non-fungible tokens (NFTs) (32).

### Problem 4. Discriminatory Biases: Rubbish in, Rubbish Out

Perhaps the most commonly discussed problem concerning AI technologies is their potential to exacerbate our current societal biases in clinical practice. Even more alarmingly, developing models with historical data may perpetuate our historical biases on a variety of different patients in ways we have since improved from.

Issues concerning discriminatory bias may manifest as a model demonstrating high performance on a single sample of patient data, but then failing on different subsets of individuals. For instance, an increasing focus in ML based applications has been to create algorithms capable of assisting dermatologists in the diagnosis and treatment of diseases of the skin (33). While racial inequalities to healthcare delivery are becoming more and more documented across the world, recent work has suggested one of the major sources of these inequalities stems from the lack of representation of race and skin tone in medical textbooks (34). Medical schools in recent years have immediately begun to address these concerns by updating textbook images for increased inclusivity, yet deep learning (DL) image-based classifier algorithms often continue to use low quality datasets for training, which commonly contain unidimensional data (e.g., mostly lighter skin images) (35). In turn, these algorithms will perform better on the image type it is trained on, and subsequently propagate biases that were represented in the original datasets. This cycle ultimately provides the chance for profound failures with specific groups of peoples. In a study examining three commercially available facial recognition technologies (Microsoft, Face++, and IBM) based on intersectional analyses of gender and race, differences in error rates of gender classification were demonstrated to be as high as 34.7% in darker-skinned females compared to 0.8% in lighter-skinned males (36). Ultimately, even if some diseases are more common in specific races or genders, such as melanoma in non-Hispanic white persons, all patients with a variety of skin types should be included for the potential benefits of these algorithms in the future (35). While these concerns may be more obvious, even the location of the image acquisition center can bias a model's performance. This is due to the demographical makeup of the surrounding community in which images were trained and tested. Thus, when the model performs in a different environment, its performance will drastically differ based on the new demographics encountered.

Improved patient related factors must be considered when building datasets for the training of a model. By building models on data from a variety of different sites with increased awareness of the specific populations included, we may begin to mitigate the potential biases in our results. Ultimately, improvements in DL algorithms remain relatively nascent, and there has been an increased focus on classification performance across gender and race, providing us with impetus to ensure that DL algorithms can be successfully used to mitigate health care disparities based on demographics (35–37).

### Problem 5. Generalization to New Populations: From the Few, but Not the Many

Expanding on concerns of discriminatory biases, problems with generalization may occur due to the expansion of global software markets. Aside from differences in gender and skin color alone, a model may fail based on datasets trained on individuals from a single population due to underestimating the differences in population driven variability.

Most consider that using multi-centric data improves the external validity of a model's results given that it tests samples of individuals from a variety of locations. However, if all the centers providing data are within a single country, such as in the United States for instance, how will these models perform in China where there are unique individual characteristics that are concomitantly shaped by differences in the environment? For example, one of the most accepted neurobiological models of language suggests a dominant left-lateralized system. However, many of these models were based on participants that speak English or are from the United States (38), while other studies including Chinese participants suggest a right-lateralized white matter system related to learning Mandarin (39). In fact, other studies have suggested these results also expand to non-Chinese subjects who learned Mandarin as a non-native language, such as European subjects (40), suggesting that differences in white matter connectivity may be more pronounced for some tonal languages. However, it is also reasonable to conjecture based on the effects of these subtle differences that there are likely additional underlying inter-individual differences not being considered in this paradigm outside of just tonal languages alone (41). Without consideration of differences across separate datasets (42), unexpected performances in ML-based brain mapping software could jeopardize market expansion into different areas outside of where the model was originally developed.

To prevent and manage issues of generalization, a number of solutions exists. First, similar to what was described above, data must be accumulated from a variety of sources. However, to provide the most generalizable results, these sources must span several different sites inside and outside of the country of origin where the model was developed. Surely, inter-individual factors must be considered during production to improve the robust ability of a model in different environments. Nonetheless, it is also likely that site-specific training should also be considered as an optimal avenue to tailor models based on the specific populations where a model is going to be implemented. Then, external validation testing in separate adequately sized datasets (43) can ensure that an algorithm can model data from different sites similarly to that which it trained on.

Importantly, improved collection of multi-site data simultaneously raises concerns of patient anonymity, patient agency and informed consent. Fortunately, a great deal of progress has been demonstrated with methods of federated learning to deal with the bias of models when trained with homogenous populations (44). Federated learning methods improves the maintainability of data anonymity when sharing patient data across numerous sites, thus allowing for improved research collaboration and model performance across heterogenous populations (44). However, given the ability for various ML systems to re-identify individuals from large datasets, a key improvement in the future suggested by Murdoch (45) will likely also include recurrent electronic informed consent procedures for new uses of data and further emphasis on the respect for the ability of patients to withdraw their data at any time.

### Problem 6. Emergence of New Trends: Surfacing Creatures From the Depth

This problem is likely the most relevant to the current conditions of the world with the recent SARS-CoV-2 pandemic. As such, problems related to the *emergence of new trends* refers to when a new trend emerges in the data that the initial model was not built to account for, thus altering the new statistical comparisons being made between variables.

Previously, ML techniques have been commonly applied to predict changes in seasonal diseases, such as influenza (46), to further allow hospitals to appropriately prepare for medical supply needs, such as bed capacity, and to appropriately update both vaccine developments and citizens themselves of prevalent circulating strains. This is because many viruses commonly mutate and produce a variety of strains each year, yet vaccines can only account for a number of the most prevalent strains. In such paradigm, ML tools can be applied to estimate which strains will be most common in upcoming seasons with high accuracy to be included in upcoming seasonal vaccines (46). However, unexpected changes can occur in the environment, such as a new pandemic, which drastically alters the environmental landscape and therefore changes the way two variables may be modeled based on new environmental parameters. If there is not an ongoing monitoring system in place, these models can lead to potential harm as results are no longer reliable. Similarly, medical devices are constantly being altered and upgraded to improve their diagnostic and visualization abilities, such as for functional magnetic resonance imaging (fMRI) scanners. However, magnetic field inhomogeneity between different scanners, such as a 3 Tesla vs. a newer 7 Tesla, could lead to differences in relative blood oxygen level-dependent (BOLD) signal intensity, and therefore contains poor inter-scanner reliability (47). As such, when utilizing brain mapping software on an individual patient with different scans, erroneous brain network anomalies may arise and can lead to inappropriate neurosurgical treatments just merely due to the inability of a model to account for differences in functional magnetic resonance imaging (fMRI) scanners utilized.

Models in production should be created with a set of test reflective environmental data to ensure expected performances *in situ*. Furthermore, alongside changes in current clinical practices, models must be continually monitored and tested with new data to assess for reliability and validity. This continual external validation testing with separate adequately sized datasets than which it was trained on provides a necessary avenue for improvement as the field of healthcare and the environment itself is continually changing.

## Method: How Does the Tech Approach Play Out?

### Problem 7. Reproducibility: Bad Copies

**Figure 5:**
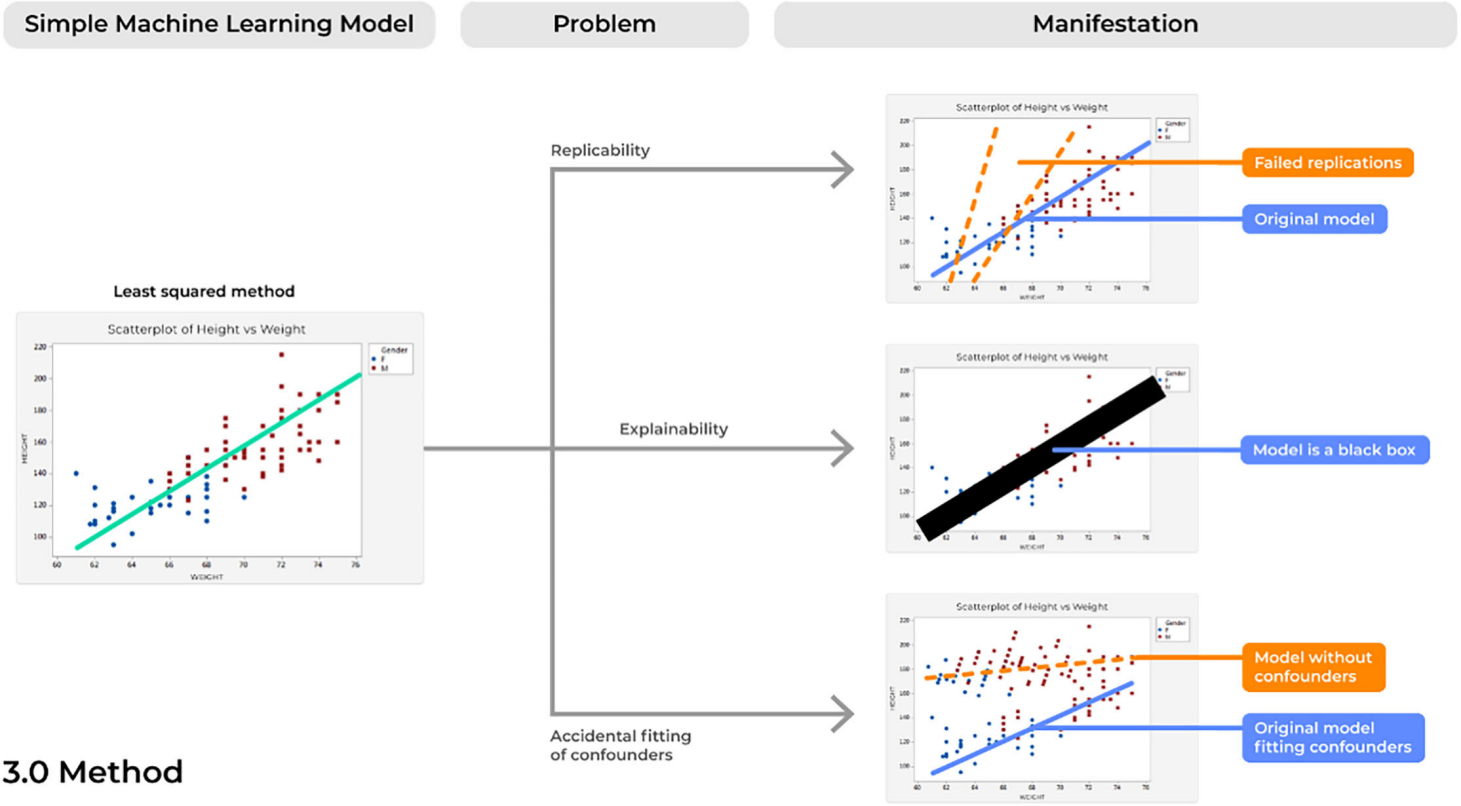
Concerns of the methodology utilized to develop an ML model.

Concerns of replicability are not a newly discussed phenomena for a number of different fields that require the processing of large amounts of data (48). However, failure of a model to demonstrate the same results time and time again presents a profound risk of inconsistencies in the delivery and quality patient care, and therefore should be considered in future ML developments (Figure 5).

To ensure an ML algorithm applied in the healthcare setting is fully reproducible, some (49) have suggested that a study should produce the same results according to (1) technically identical conditions (related to code and dataset release), (2) statistically identical conditions (related to differences in sampled conditions still yielding the same statistical relationships), and (3) conceptually identical conditions (related to how the results are reproduced in accordance with pre-defined descriptions of the model's effects). When these methods of reproducibility are not met, the one who created the model would be unable to replicate its results on subsequent runs. Furthermore, when others are attempting to assess the model, possibly to improve its applicability, they too will be unable to obtain the reported effects by the original authors. As such, recent methodological and reporting standards have been proposed to address these issues, such as the Transparent Reporting of a multivariable prediction model for Individual Prognosis OR Diagnosis (TRIOPD), and its recent statement for ML-prediction algorithms (TRIPOD-ML) (50, 51).

In addition to the obvious potential improvements in patient safety following more rigorous evaluations of clearly reported methodology, improved reporting of ML algorithms can also provide an important way to advance the field as a whole. A number of different Data Scientists may spend countless hours in designing complex ML models to address an imminent question, such as what we saw for COVID-19, yet increasing errors will commonly be identified from just the smallest differences across algorithms (8). Instead, if models and datasets are clearly reported following a study, then others can appropriately assess these models and collectively improve upon them to produce more robust pipelines. This will ultimately improve our ability to bring these tools to clinical practice as a model becomes more accurate without repeating the same mistakes. The increased requirements for adherence to rigorous, ML reporting guidelines across many major peer-reviewed journals is a promising improvement moving forward.

### Problem 8. Explainability: The Black Box Problem

One of the largest concerns of AI-based devices in medicine concerns physicians' lack of trust for model performance (52). Unfortunately, as ML models have increased in complexity, this improvement has often been met with a trade-off in explainability, in which there is increasing uncertainty regarding the way these models actually operate (53).

This problem is often described as a model operating in a “black box,” in which irrespective of model performance, very little can be elucidated about why a model made a specific decision. A common example of this can be seen with a highly powered ML technique known as deep learning (DL). DL applications can maintain hundreds of stacked representations across hundreds of layers, a relationship that no human can truly accurately comprehend in full detail. However, a number of important improvements can be made in the field as we improve concerns of lack of explainability, to which a whole field has been dedicated known as Explainable Artificial Intelligence (XAI) (53). Ultimately, ML tools are capable of taking highly dimensional data and quickly making accurate decisions in highly time-critical medical scenarios, a feat that humans may never physically nor cognitively be capable of performing (54). However, if we could explain the various decisions being executed by a certain model and the specific features being analyzed to produce a certain outcome (24), physicians can better interpret these results based on logic and previous knowledge. Then, healthcare providers may not only be able to better trust these algorithms, but providers may also continually improve the model's performance when the system presents an error that is likely based on a specific wrong answer possibly being executed in a portion of a decision tree. In fact, since these models are highly capable of detecting novel patterns in large amounts of data which are invisible to the human eye (4), interpretable and explainable models may also unlock new insights in the scientific world that spur further improved ML developments in the future, creating a positive reinforcing cycle of innovation.

Outside of the trust of a practicing healthcare provider, the patient themself, if diagnosed by a ML tool to have a malignant skin lesion, may too require an interpretable and justifiable reason why specific results were provided, such as *why* the tumor was diagnosed as malignant. Thus, it is important that a clinician is able to interpret the decisions made by a specific algorithm, but this also raises concerns of violating patient-physician relationships and liability for AI technology in general (55). A core component of the Hippocratic Oath requires physicians to do no harm and act upon their greatest judgement for improved patient care. With the incorporation of machine-learning guided clinical care, failure to understand a model's decision making can shift fiduciary duties away from the physician and hurt the patient-physician alliance (56). Furthermore, if a model provides a piece of information that leads to a poor outcome for a patient, is it the *machine's* fault or is it the *healthcare provider's* medical error? Unsurprisingly, promotion of interpretability of a model is outlined as a main principle within the recent World Health Organization (WHO) guidelines on *Ethics & Governance of Artificial Intelligence for Health* (57). Both the model and provider must be able to clearly elucidate these findings to the patient if we are to truly incorporate ML into standard medical practices.

Movement toward white-box, also called glass-box, models provides a solution to address concerns of explainability. These models can often be seen with linear (58) and decision-tree based models (24), although a number of other applications are increasingly being developed (53). In fact, DL based networks make up the majority of the highly sought after radiological-AI applications for the medical field (1), such as the systems that can diagnose brain cancer during surgery. Such networks provide the enthusiasm for the recent large scale efforts in the field to improve the explainability of advanced ML techniques (59). Specifically, by utilizing white box models as first line modeling techniques, one can ensure findings are being appropriately made based on ground truths provided by current scientific knowledge. For example, a number of recently developed practical approaches have been introduced using input vector permutation to better understand how specific inputs impact the predictions of a model and may be particularly useful to gain insight into how models make specific decisions (60, 61). Explainable AI approaches, such as deconvolution methodology, can be applied to more complicated models, such as convolutional neural networks (CNNs) and ensembles, to improve the interpretability of the more complex models (62). However, further research is needed in the field of explainable AI to better understand model-specific techniques that can be leveraged to ultimately improve the transparency of these models in the healthcare setting.

### Problem 9. Accidental Fitting of Confounders: Guilt by Association

ML tools are able to digest highly complex datasets by continually assessing and scanning different features until optimal performance is achieved. As such, concerns of accidentally fitting confounders can easily surface and a model that was thought to be capable of predicting an outcome is instead making a prediction based on factors unrelated to that outcome of interest. If so, these models can produce not only unreliable results in clinical practice, but can also present profound risks of patient harm, such as by under- or over-estimating specific diagnoses.

An example of this problem can be seen with a model that is purported to show great performance in detecting autism. However, if not carefully assessed for confounders, one may miss that the model is actually detecting head motion. For instance, patients with autism often move more in fMRI scans and this can cause head motion artifacts that compromise fMRI data due to altering voxel and stable state magnetization. Ultimately, this will cause scans to show false regions of increased/decreased brain activity that are misused to diagnose autism (63). If head motion is not corrected for, the performance of these models will collapse (64). Unfortunately, these children may have already received unnecessary treatments (65) that resulted in increased financial burden (66) and possibly decreased treatments for other diagnoses (67). Alarmingly, the literature presents a number of additional examples of this problem that have may have gone unnoticed in certain ML algorithms.

First, an ML specialist must have a strong understanding of the data being modeled. Then, when actually developing the model, one should carefully explore and rule out any concerns for confounders. In addition to previous descriptions of “white-box” models, improved understanding of the features being mapped may allow further appropriate critical evaluations of model performances and in turn lead to increased trust in the medical community.

## Operation: In the Field

### Problem 10. Model Drift: Like a Rolling Stone

**Figure 6:**
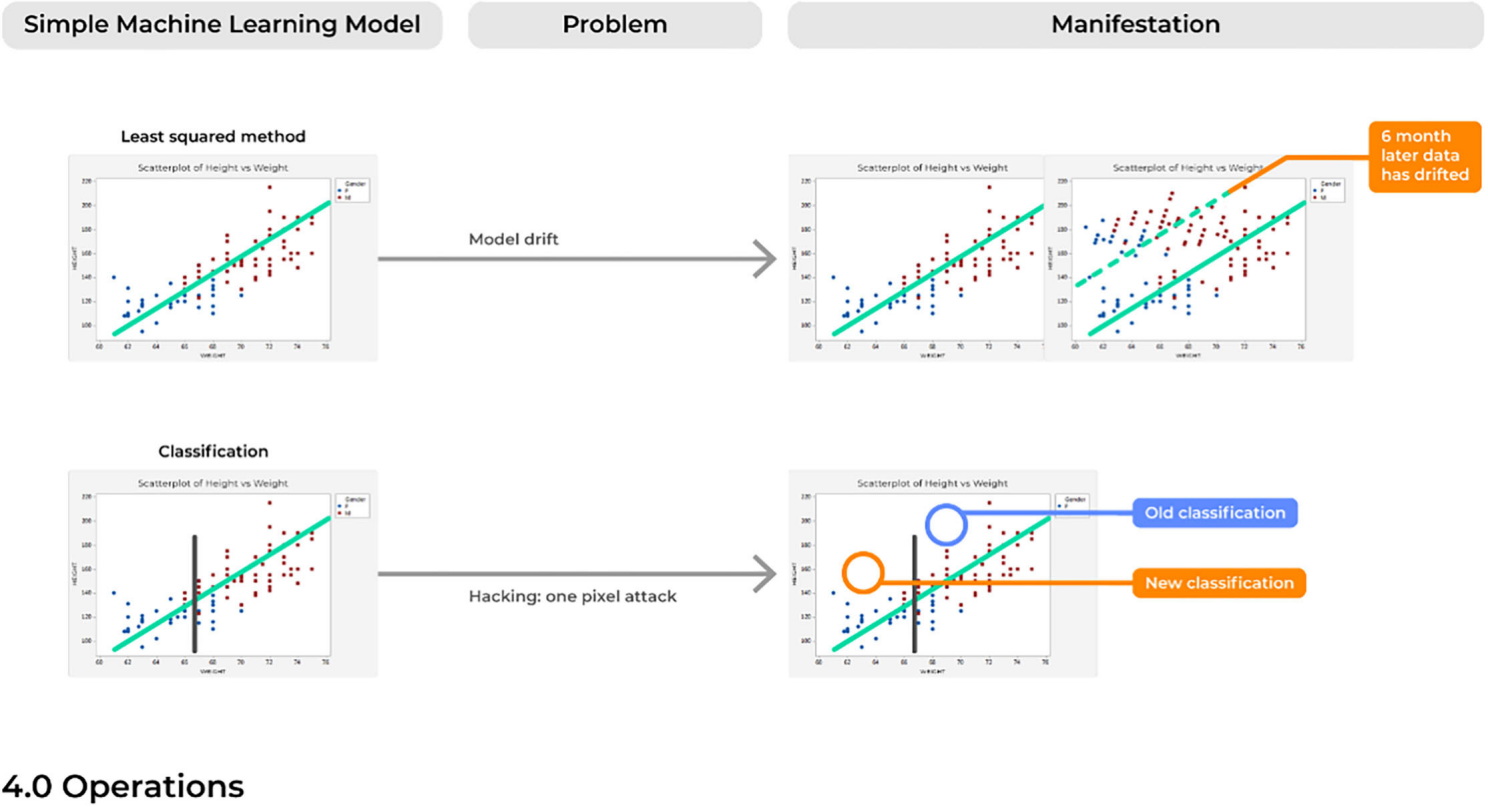
Concerns with the operations of an ML-system.

For many of the reasons discussed above, over time a model will likely begin to make an accumulating number of errors. This could be due to issues with *model drift*, in which a model that was deployed into production many years ago would begin to show performance decay over time (Figure 6). Different than problems with *the emergence of a new trend, model drift* represents a multifactorial issue that likely reflects the relationship between two variables changing with time, ultimately causing a model to become increasingly unstable with predictions that are less reliable over time.

Generally, the training of ML models follows an assumption of a stationary environment; however, two types of model drift based on non-stationary environments have been described, including: (1) virtual concept drifts and (2) real concept drifts (68). Virtual drifts refer to when the statistical characteristics or marginal distributions of the actual data changes according to a change in time without the target task itself also adjusting similarly (e.g., the regression parameters). Real drifts refer to situations when the relationships between two or more variables in a model are based on a function of time, such that parameters in which the model was trained now becomes obsolete at different points in time (e.g., Pre-Covid vs. Post-Covid) (69). Without considering the possibility of a model drifting, a model can begin to predict outcomes in an unexpected way, which in a healthcare setting could immediately represent incorrect diagnoses being made.

To account for model drift, both active and passive methods have been proposed, of which the later represents the easiest solution to implement (68). Active methods refer to the methodology for detecting this drift and then self-adjusting its parameters to retrain the system to account for this shift, such as by forgetting old information and then updating based on new data (70). However, this methodology is more practical when data is available as a continual stream that will allow a model to continually adapt to recent data inputs. Differently, passive learning methods are reliable in that the performance of a model will be continually or periodically monitored by developers, such as through each release cycle, thus ensuring consistent and reliable results according to the model's original results. As more data becomes available, passive methods could allow users to adapt the model and retrain it based on new data and updated scientific knowledge. Thus, this method could allow for more transparency over time concerning the model's performance, avoiding scenarios where a model may make decisions on new relationships that are non-interpretable or even scientifically unsound.

### Problem 11. Practicality Over Hospital Context: Will the IT Department Say Yes?

Systems should be developed according to the environment in which they will be deployed. While this may seem intuitive, there are a number of strict requirements that technology must follow in a healthcare setting that may not be accounted for, especially with cloud-based computing software. Thus, a system should be developed based on how hospitals are organized and specifically how healthcare providers will plan to use these models.

A key concern can be seen with the Health Insurance Portability and Accountability Act of 1996 (“HIPAA”) (71)–as well as other patient and individual privacy standards across the globe. Only those who require the handling of patient of data at a given time for the ultimate care of the patient are permitted access to patient data. Therefore, a cloud-based computing system that leaks data to the cloud presents a clear violation. This is not to say cloud-based software cannot be used in medicine given that internet-based methodology demonstrates several beneficial ways to increase the capacity of a hospitals operating system. In fact, current EHR systems represent the standard for the digital storage, organization, and access to healthcare records, and thus cloud-based computing will likely become standard IT infrastructure in the future. Nonetheless, specific rules and regulations must be considered prospectively to adjust a specific system to the HIPPA and IT requirements of a given healthcare system (72, 73). Furthermore, as mentioned previously, if the system implemented is too computing heavy, the model itself may become impractical as it can take hours to run on a less-powered healthcare provider's laptop.

To consider impending concerns of meeting the rigorous standards and requirements contained in the hospital context, developers should meet with the end users and product stakeholders at the beginning of production. In turn, this will allow a clear delineation of the current restraints of an environment that will allow developers to prospectively include user requirements in solution designs.

### Problem 12. Hacking: One Voxel Attack

Despite the novelty of advanced ML systems that are highly capable of managing complex data relationships, it must be remembered that ML systems are inherently IT systems which can be similarly fooled and hacked by outsider users.

One of the most common applications of AI is for image classification of radiologic scans. Deep neural networks are highly capable of analyzing imaging scans, allowing them to determine if a scan presents an image of a malignant or benign tumor (1) or can even differentiate between different types of highly malignant tumors often within a time frame unimaginable for humans. Nonetheless, the ability to fool AI models is a long-understood threat, possibly accomplished just by rotating the imaging scan (74). One particular well-known threat is described as the “one-pixel attack,” referring to the ability to drastically fool a neural network by just changing a single pixel in the image being analyzed (75). In turn, this causes the model to classify the image as being of a different class than what is actually represented in the image. Ultimately, this single form of hacking merely suggests the vulnerable nature of ML systems, and also contributes to the truth that we do not always fully understand how a model may be working. Therefore, when a model is failing, we may not be fully aware of this failure. As such, there are profound concerns of similar cyber attacks on ML software in the medical field, especially given the often mere dichotomous classifications asked for by providers with these image-based classification methods (e.g., malignant or benign). Such attacks also present enormous danger to the field of AI itself, which following an attack–could spur long-periods of mistrust with the medical community.

A number of methods have been proposed to prevent the damage from these adversarial attacks. Re-training the model with robust optimization methodology can increase the resistance of a model to these attacks. Increase detection methods to identify attacks may also be appropriate (76). Other methods have also been similarly described, but it remains uncertain the degree to which these methods are better than others for a given scenario. Nonetheless, what is certain is that the integrity and robustness of an AI system must be rigorously examined against known attacks to achieve further safety and trust with applications in the medical field.

## Future Directions: a Standard Labeling System for Medical AI

Addressing each of the concerns above provides a way to rigorously create a robust model that performs safely and accurately in a field full of potential concerns. However, one way to further advance the field and improve the widespread adoption of these robust technologies is through a standard labeling system that can accurately detect and then convey anomalies in an ML-based system's performance and quality (77).

**Figure 7:**
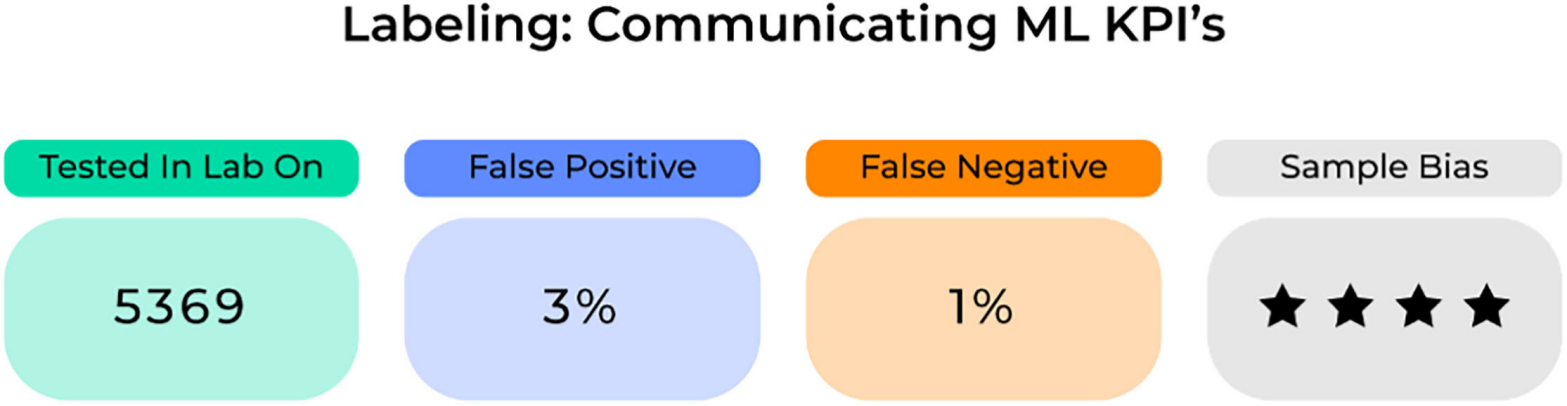
Standard labeling system according to Key Performance Indicators (KPIs).

Common to the most successful business plans are the use of key performance indicators (KPIs) as a way to document success and efficiency. KPIs demonstrate the achievement of measurable landmarks toward reaching company and consumer goals. For an ML model, standard labeling could display KPIs possibly related to the (1) sample it trained and tested on, (2) its quantitative accuracy including information on false positive and negatives, and (3) its risk of specific biases (Figure 7). Importantly, these KPIs need to be clearly defined and continuously updated in order for healthcare providers to appropriately examine and incorporate specific ML-based systems into standard clinical practices. Ultimately clinicians will need to assess a model's success to understand where and when to apply it in a given scenario. To make this decision, it will require the use of accurate performance metrics for each model on the target population.

An important concern where standard medical labeling may be of use is in determining medical liability. While it is justified to create incentives for risk control based on the environment of application, the degree of responsibility is less clear cut, and who is ultimately responsible: the *machine* or the *healthcare provider*? While the ethics of AI are outside the scope of this paper, further objective information on a model's performance for a given population with a standard labeling system, possibly contained in the legal section of an ML system, will ultimately improve our objective insight into their performance abilities. In turn, this can give healthcare providers more complete information on whether or not to incorporate these advanced systems in specific scenarios or not.

## Conclusion

Advancements in the field of artificial intelligence have promised a number of exciting and promising applications for the medical field to improve the quality and delivery of health care services. While there have been remarkable advances in previous years, these applications have yet to fully demonstrate their true potential in clinical applications due to failures in demonstrating reproducible and reliable results as well as the general mistrust of these technologies in the medical community. We outline many of the problems that future developers will likely face that contribute to these failures, specifically related to the approach, data, methodology, and operations of machine learning based system developments. If we continue to prospectively address and manage these concerns with reliable solutions and appropriate system processes in place, then we as a field may further optimize the clinical applicability and adoption of medical based AI technology.

## Author Contributions

SD: writing, reviewing, editing, and conceptualization. ND: writing, reviewing, and editing. Both authors contributed to the article and approved the submitted version.

## Conflict of Interest

SD is a co-founder, chief data scientist, and shareholder for Omniscient Neurotechnology. The remaining author declares that the research was conducted in the absence of any commercial or financial relationships that could be construed as a potential conflict of interest.

## Publisher's Note

All claims expressed in this article are solely those of the authors and do not necessarily represent those of their affiliated organizations, or those of the publisher, the editors and the reviewers. Any product that may be evaluated in this article, or claim that may be made by its manufacturer, is not guaranteed or endorsed by the publisher.
